# Insights into Aflatoxin B1 Toxicity in Cattle: An In Vitro Whole-Transcriptomic Approach

**DOI:** 10.3390/toxins12070429

**Published:** 2020-06-29

**Authors:** Marianna Pauletto, Roberta Tolosi, Mery Giantin, Giorgia Guerra, Andrea Barbarossa, Anna Zaghini, Mauro Dacasto

**Affiliations:** 1 Division of Pharmacology and Toxicology, Department of Comparative Biomedicine and Food Science, University of Padua, Viale dell’Università 16, I-35020 Legnaro (Padua), Italy; marianna.pauletto@unipd.it (M.P.); roberta.tolosi@studenti.unipd.it (R.T.); mery.giantin@unipd.it (M.G.); giorgiaguerra94@gmail.com (G.G.); 2 Department of Veterinary Medical Sciences, University of Bologna, Via Tolara di Sopra 50, Ozzano dell’Emilia, 40064 Bologna, Italy; andrea.barbarossa@unibo.it (A.B.); anna.zaghini@unibo.it (A.Z.)

**Keywords:** Aflatoxin B1, cattle, aflatoxicosis, mycotoxins, liver, fetal hepatocyte cell line, RNAseq, transcriptome, cancer

## Abstract

Aflatoxins, and particularly aflatoxin B1 (AFB1), are toxic mycotoxins to humans and farm animal species, resulting in acute and chronic toxicities. At present, AFB1 is still considered a global concern with negative impacts on health, the economy, and social life. In farm animals, exposure to AFB1-contaminated feed may cause several untoward effects, liver damage being one of the most devastating ones. In the present study, we assessed in vitro the transcriptional changes caused by AFB1 in a bovine fetal hepatocyte-derived cell line (BFH12). To boost the cellular response to AFB1, cells were pre-treated with the co-planar PCB 3,3′,4,4′,5-pentachlorobiphenyl (PCB126), a known aryl hydrocarbon receptor agonist. Three experimental groups were considered: cells exposed to the vehicle only, to PCB126, and to PCB126 and AFB1. A total of nine RNA-seq libraries (three replicates/group) were constructed and sequenced. The differential expression analysis showed that PCB126 induced only small transcriptional changes. On the contrary, AFB1 deeply affected the cell transcriptome, the majority of significant genes being associated with cancer, cellular damage and apoptosis, inflammation, bioactivation, and detoxification pathways. Investigating mRNA perturbations induced by AFB1 in cattle BFH12 cells will help us to better understand AFB1 toxicodynamics in this susceptible and economically important food-producing species.

## 1. Introduction

Aflatoxin B1 (AFB1) is a secondary metabolite of fungi (mostly *Aspergillus flavus* and *A. parasiticus*) [1]. It can contaminate both feed (cereals and oil-rich agricultural crops) and food (e.g., meat, milk, eggs), causing various diseases and health issues in humans and farmed animals. Aflatoxin B1 is the most toxic aflatoxin, having hepatotoxic, immunotoxic, mutagenic, carcinogenic, and teratogenic properties. Strong evidence linking AFB1 consumption with hepatocellular carcinoma (HCC) occurrence led the International Agency for Research on Cancer (IARC) to classify this mycotoxin as a Group I carcinogen for humans [2]. Noteworthy, several studies have also demonstrated a strong, synergistic interaction between AFB1 exposure and chronic hepatitis C virus infection in humans, e.g., [3].

In farm animals, AFB1 may cause a plethora of untoward effects, leading to decreased animal growth and productivity [4,5,6,7,8], commonly referred as aflatoxicosis. As a consequence, livestock exposure to AFB1-contaminated feed can lead to significant economic losses [9,10]. These effects are not only species-specific, they also depend on the animal’s individual susceptibility, the level of aflatoxins contamination, and the duration of the exposure [11]. Thus, studying the effects of AFB1 in all livestock species and in different animal production systems (e.g., diverse production sectors, i.e., dairy or beef cattle, broilers or lying hens) appears of fundamental importance.

All animal species are susceptible to aflatoxicosis, but outbreaks occur most frequently in pigs, sheep, and cattle [12]. Noteworthy, it is also known that beef and dairy cattle are more susceptible to aflatoxicosis than sheep or horses. Poultry are sensitive to AFB1, the order of sensitivity is ducks > turkeys > Japanese quail > chickens [13]. As to aquatic species, trout is the most sensitive species to AFB1 [14]. These differences are usually attributable to species differences in AFB1 metabolism and detoxification and, specifically, to the constitutive expression and catalytic activity of key enzymes involved in these mechanisms [15,16]. For instance, the extreme sensitiveness of poultry species to AFB1 appears to be a consequence of the high activity of phase I drug metabolizing enzymes (involved in AFB1 bioactivation) and a deficiency of key conjugative enzymes [17]. Interestingly, whole-transcriptome studies in turkeys proved these mechanisms are also responsible for the different susceptibility to AFB1 between wild and domesticated animals (e.g., [18,19]).

In all animal species, AFB1 is bioactivated by cytochrome P450 (CYP) enzymes in the liver; in particular, CYP1A and CYP3A isoforms produce different toxic metabolites, including AFB1-exo-8,9-epoxide (AFBO), AFM1, aflatoxicol (AFL), AFB2a, AFQ1, and AFP1 [15]. The epoxide derivative is the most toxic metabolite of AFB1, as it may bind to guanine residues of nucleic acids. These adducts can induce DNA mutations and inhibit DNA transcription and RNA translation [20,21]. This harmful metabolite can be partly hydrolyzed to AFB1-dihydrodiol either spontaneously or through a reaction catalyzed by epoxide hydrolase (EPHX). Nevertheless, this metabolite can bind lysine residues, leading to protein damage and necrosis, thus representing an additional AFB1 toxic mechanism [22,23]. The major AFBO detoxification pathway is represented by the conjugation with glutathione, catalyzed by glutathione-S-transferase (GST), particularly the GSTA1 isoform. This reaction converts the reactive metabolite in polar and less toxic derivatives excreted with the urine [24].

A further AFB1 microsomal biotransformation is the CYP1A2-dependent hydroxylation to AFM1, a metabolite commonly detected in humans and animals exposed to AFB1. This derivative is the most carcinogenic AFB1-hydroxylated derivatives because, likewise to AFBO, it is able to form DNA adducts [25]. Accordingly, AFM1 has been classified as a group 1 carcinogen by IARC [26]. This metabolite may be conjugated with glucuronic acid by uridine 5′-diphospho-glucuronosyltransferases (UGTs) and, subsequently, excreted via the bile. Alternatively, unmodified AFM1 is excreted through urine or milk [27,28,29,30]. Because of its excretion in milk, AFM1 contamination in dairy products is a serious concern, as it might pose severe health risks for humans in general and for susceptible population groups such as infants and young children [28,31]. Hence, the full characterization of AFB1 mechanistic toxicology in dairy cattle, together with the possible tools to reduce its consequences in humans in animals and humans, is gaining increasing interest in the scientific community.

As far as the relationship between AFB1 and carcinogenesis is concerned, AFBO represents the metabolite mostly involved in the genotoxic process (i.e., DNA mutations). A great number of epidemiological studies on HCC patients exposed to high levels of aflatoxins proved that some genes (e.g., P53 tumor suppressor gene, c-KRAS oncogene, and HRAS proto-oncogene) are particularly subjected to these mutations [32,33,34,35], strengthening the association between HCC and aflatoxin exposure. Additional mechanisms have been suggested to contribute to AFB1 carcinogenesis: AFB1 exposure has been associated with rising oxidative stress and the resulting generation of reactive oxygen species (ROS) [25,36] and lipid peroxidation. The former may react with DNA, causing strand breaks and mutations, which could initiate carcinogenesis [37]. As to lipid peroxidation, it inhibits DNA repair and triggers the production of an adduct, i.e., the cyclic -methyl-hydroxy-1,N2-propano-dG (meth-OH-PdG) [38].

Transcriptional profiling is a widely used tool to investigate the mechanistic toxicology of mycotoxins [39]. Some in vitro studies have explored the transcriptomic alterations associated with AFB1 exposure, and most of these studies have been conducted in both human primary hepatocytes and hepatoma cell lines (i.e., HepaRG and HepG2) [40,41,42]. Overall, hepatoma cell lines showed a certain differential response to AFB1 (24 or 72 h of incubation). However, most of the common differentially expressed genes (DEGs) belonged to pathways related to DNA damage (e.g., p53 and apoptosis), biotransformation (e.g., CYPs), and receptor-mediated processes [32]. In an additional study, Smit and colleagues demonstrated how AFB1 modulated the expression of several key transcriptional factors, being P53 and the aryl hydrocarbon receptor (AHR) two of the most relevant ones [42]. Finally, a strong enrichment in genes associated with cell cycle, cellular movement, assembly and organization, cell growth and proliferation, as well as DNA replication and repair was observed in HepaRG exposed to AFB1 for 72 h [40].

While whole transcriptional changes attributable to AFB1 toxic mechanisms have been investigated in human hepatocytes, scarce information is available in susceptible livestock species. Some RNA sequencing (RNA-seq) studies, aiming at investigating liver transcriptional changes elicited by AFB1, have been conducted in turkey and ducklings [6,43]. In the former species, DEGs were somewhat linked to cancer, apoptosis, cell cycle, drug metabolism, inflammation, or lipid regulation [6,44]. Likewise, in ducklings AFB1 triggered a transcriptional response involving genes of oxidative and conjugative drug metabolism, carcinogenesis, apoptosis, cell cycle, and fatty acid metabolism [43].

To our knowledge, whole-transcriptomic studies investigating the molecular effects of AFB1 in cattle liver have never been performed so far. Based on these considerations, the aim of the present study was to assess in vitro the overall transcriptional changes (RNA-seq) caused by AFB1 in a bovine fetal hepatocyte-derived cell line (BFH12). This cell line constitutively expresses most cattle drug metabolizing enzymes and transporters [45,46], but its transcriptome has not been fully characterized so far. With the intent to have a greater response of the aforementioned in vitro model, cells were pre-treated with the co-planar polychlorinated biphenyl 3,3′,4,4′,5-pentachlorobiphenyl (PCB126), a known agonist of the aryl hydrocarbon receptor (AHR) [47]. Investigating mRNA perturbations induced by AFB1 in the cattle BFH12 cell line will help us to better understand AFB1 toxicodynamics in this susceptible and economically important food-producing species. These molecular data might help in developing or addressing strategies to mitigate aflatoxin effects in dairy cattle, thus avoiding significant economic losses and reducing the risk of dairy product contamination. In perspective, it could be interesting to test if natural feed additives/byproducts (e.g., polyphenols) might reduce the detrimental effects of AFB1 in this cell model, and which biological processes are involved in these mechanisms.

## 2. Results

### 2.1. Preliminary Evaluations of BFH12 Cell Responsiveness to PCB126

BFH12 cells exposed for 24 h to PCB126 exhibited a very low cytotoxicity (approximately 6%) and only at the highest tested concentration (i.e., 100 nM; data not shown).

PCB126 was demonstrated to upregulate the mRNA expression of some members of the “AHR gene battery” [48] even at a concentration of 1 nM. Among the three selected time points (6, 12 and 24 h), the maximum effect was reached at 24 h, being CYP1A1, CYP1B1, and AHRR the most relevant genes (Figure S1). Therefore, this latter concentration and the 24 h time point were chosen for the pre-treatment of BFH12 cells in the following experiments.

To verify if the pre-treatment with 1 nM PCB126 for 24 h might increase AFB1 cytotoxicity, the WST-1 assay was carried out on cells treated with AFB1 (for 24, 48, and 72 h of incubation) with or without pre-incubation with PCB126. Cell pre-treatment with PCB126 markedly increased AFB1 cytotoxicity, significantly reducing the IC_50_ value (Figure S2).

### 2.2. Aflatoxin B1 Cytotoxicity

**Figure 1:**
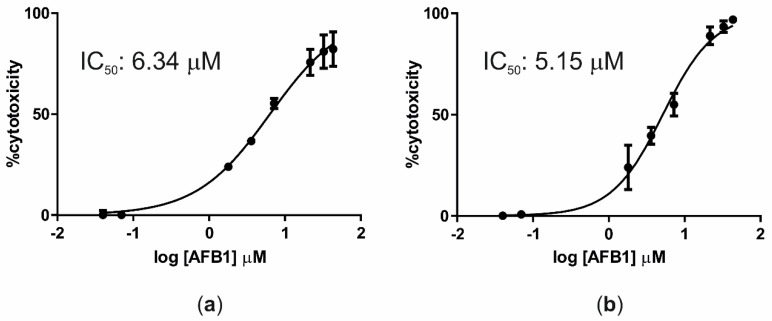
Dose–response curves (cytotoxicity) of AFB1 after incubation of BFH12 cells for 48 h (**a**) and 72 h (**b**). Graphs were obtained by means of GraphPad prism software and using three independent cell culture experiments, each one run in sextuplicate. Data are expressed in mean cytotoxicity rate ± mean standard error (SEM).

Cells exposed for 24 h to increasing AFB1 concentrations exhibited a low mortality rate; therefore, it was not possible to build a dose–response curve and define the corresponding IC_50_ value. However, after 48 and 72 h of incubation with the same AFB1 concentrations, IC_50_ values of 6.34 μM (R^2^ = 0.97) and 5.15 μM (R^2^ = 0.96) were obtained, respectively (Figure 1).

### 2.3. Aflatoxin B1 Biotransformation in BFH12 Cells

BFH12 cells were proved able to metabolize AFB1, producing and releasing into the medium its foremost derivatives, i.e., AFM1 and AFL (Table 1). As expected, AFM1 and AFL were not detected in cellular pellets; however, the amount of cellular AFB1 was low.

### 2.4. Whole-Transcriptome Differential Expression Analysis

A total of 236,040,833 raw reads were obtained and deposited in GeneBank under the BioProject accession PRJNA627332. All samples passed quality control measures for raw sequenced reads. After trimming and rRNA removal, an average of about 26 million reads per sample were retained, with ~99% of reads mapping to the *B. taurus* reference genome. Numbers of raw reads passing the filters and number of filtered reads mapping to the cow genome are provided in Table S1. The plot MDS (Figure S3) provides an unsupervised clustering of samples. The first dimension (x axis) clearly separates AFB1 from DMSO and PCB126 libraries.

The Differential Expression (DE) analysis between cells pre-treated with PCB126 and those exposed only to DMSO (vehicle) identified a total of eight genes with significantly higher mRNA levels (FDR < 0.05). These genes are reported in Table 2. The genes most upregulated by PCB126 were CYP1A1, CYP1B1, and a novel gene not annotated but having high similarity with AHRR (ENSBTAG00000026527). Additional genes were ABCB1 (i.e., multidrug resistance protein 1), one of the most important efflux drug transporters, and the transcription factor FOXQ1, known to be regulated in an AHR/ARNT-dependent manner.

**Figure 2:**
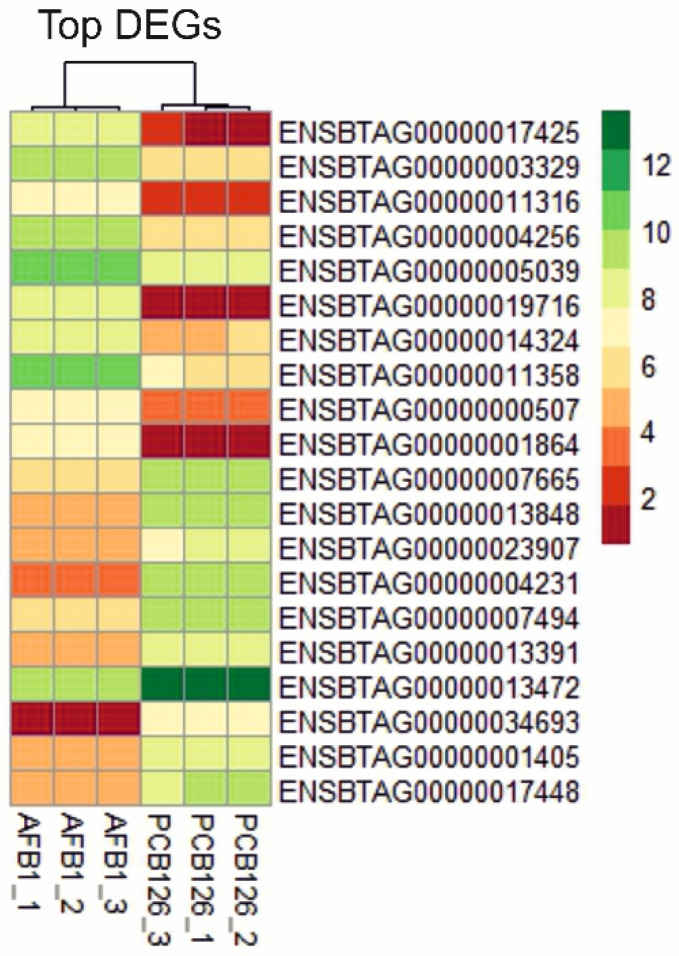
Heatmap of the top-ten genes up- and downregulated by AFB1 exposure. The graph was constructed in R environment using the pheatmap package and using as input the normalized log_2_CPM (counts per million).

To distinguish the transcriptional effects of AFB1, a DE analysis was executed between cells pre-treated with PCB126 and exposed to 3.6 μM AFB1 vs those only pre-treated with PCB126. A total of 6040 DEGs (22.7% of the entire cow genome) were identified, of which 2,632 and 3,408 were up- and downregulated, respectively. The top-ten AFB1 up- and downregulated genes are listed in Table 3 and graphically represented in a heatmap (Figure 2), while the complete list of DEGs, as resulted from EdgeR analysis, is reported in Table S2.

The most significantly over-expressed gene was GJB2, encoding for a gap-junction protein. Among the top-ten upregulated DEGs we also found genes involved in cancer, such as FST (a potential marker of HCC), ODC1 and ARAF (both considered associated with tumor promotion), and NR4A1 and NR4A3 (members of the steroid-thyroid-retinoid receptor superfamily of transcription factors, positively regulated by oncogenic signaling pathways). Even a major mediator of the inflammatory response like CXCL8, or interleukin 8 (IL8), was among the upregulated genes with the highest fold change (FC).

As regards the top-ten downregulated genes, three of them (i.e., ADGRD1, NPR3, GPM6A) were involved in G-protein coupled receptor (GPCR) signaling pathways, including those attributable to HCC; another one (GPM6A) may contribute to the regulation of endocytosis and intracellular trafficking of GPCRs. Two more (COL18A1 and COL1A2) were collagen genes possessing inhibitory effects on cancer cell proliferation [49,50]. Likewise, SMARCA2, GLCE, and EFEMP1 showed tumor-suppressor [51,52] and anti-proliferative activities [53].

In AFB1 mechanistic toxicology, a pivotal role is played by CYPs and GSTs, but the overall antioxidant response should not be forgotten. Looking at the whole list of DEGs (Table S2) and focusing on CYP isoforms, it was found that their mRNA levels were either up- or downregulated after AFB1 exposure. Notably, CYP26B1, CYP27B1, and CYP3A4 (herein called CYP3A28, the orthologue of human CYP3A4, as demonstrated in [54]) were over-expressed, while nine other isoforms, including CYP1A1 and CYP1B1, were downregulated (Table 4). GSTs, a major class of conjugative drug metabolizing enzymes, were also significantly affected by AFB1 treatment. A total of 13 GSTs were listed among DEGs (Table 5); the GST omega 1 (GSTO1, log_2_FC = −1.1) and GST pi 1 (GSTP1, log_2_FC = −1.13) were upregulated by AFB1, whereas all remaining subclasses were downregulated by mycotoxin, including all the microsomal GSTs (MGST1, MGST2, and MGST3).

As to those genes known to play a role in the oxidative stress response, AFB1 downregulated NAD(P)H quinone dehydrogenase 1 (NQO1, log_2_FC = −2.4), the nuclear factor erythroid-derived 2-like 2 (NFE2L2, log_2_FC = −0.93), and the glutathione peroxidase 7 (GPX7, log_2_FC = −1.82). On the contrary, AFB1 induced the expressions of glutathione peroxidase 1 (GPX1, log_2_FC = 1.58) and superoxide dismutase 2 (SOD2, log_2_FC = 2.57).

### 2.5. Gene Set Enrichment Analysis

The enrichment analysis of transcriptional changes, carried out by using a Gene Set Enrichment Analysis (GSEA) approach, enriched 19 and 13 hallmark gene sets (GS) among the transcripts that were up- and downregulated by AFB1, respectively (AFB1 vs PCB126; Table 6).

Concerning the GSs enriched from AFB1-upregulated genes, some of them were related to cancer development and regulation; it is the case with MYC and KRAS oncogenes (i.e., “MYC targets v1”, “MYC targets v2”, “KRAS signaling up”) or P53 tumor suppressor (“P53 pathway”). Additional GSs putatively involved in cancer were those implied in cell cycle regulation (“E2F targets”, “G2M checkpoint”) and cell proliferation (“IL2 STAT5 signaling”, “mTORC1 signaling”). Several genes induced by AFB1 treatment appeared to be linked to a cellular stress condition, as demonstrated by the significant enrichment of GS such as “DNA repair”, “Unfolded protein response”, “UV response up”, “Apoptosis”, and “Hypoxia”. Aflatoxin B1 also triggered inflammatory processes most likely mediated by TNF-α and IFN-γ (“TNFa signaling via NF-kB” and “Interferon gamma response”, respectively).

Looking at GS enriched from AFB1-downregulated genes, a significant enrichment was observed for a GS related to cancer (“KRAS signaling dn”), which includes genes classically downregulated following KRAS activation. Transcripts involved in cell–cell junctions were also significantly over-represented, as demonstrated by the enrichment of the GS “Apical junction”. Moreover, a number of genes participating in key cellular events were inhibited by AFB1, with a resulting significant enrichment of GS like “Myogenesis”, “Proteins secretion”, “Bile acid metabolism”, and “Angiogenesis”.

### 2.6. Functional Enrichment Analysis

The functional enrichment analysis performed on the list of DEGs upregulated by AFB1 identified 39 enriched Biological Processes (BPs) and 14 enriched KEGG pathways (Table S3). The same analysis carried out on DEGs downregulated by AFB1 highlighted a significant enrichment of 36 BPs and 21 KEGG pathways (Table S3).

The two BPs most significantly enriched from the list of upregulated genes were related to the mRNA translation of the 13 mitochondrial proteins involved in oxidative phosphorylation, i.e., the “mitochondrial translational elongation”, with a Fold Enrichment (FE) of 3.53 and including 55 genes, and the “mitochondrial translational initiation” (FE 3.34, 52 genes).

Overall, the enriched BPs included genes implicated in mRNA preparation (e.g., “mRNA processing”, FE 1.85, 32 genes), splicing (e.g., “spliceosomal snRNP assembly”, FE 3.57, 17 genes; “RNA splicing”, FE 1.77, 23 genes), and ribosomal activity (e.g., “rRNA processing”, FE 3.15, 30 genes). Likewise, the two most significantly enriched KEGG pathways were “ribosome biogenesis in eukaryotes” (FE 3.39, 52 genes) and “RNA transport” (FE 2.09, 65 genes).

Thinking specifically about AFB1 mechanistic toxicology, a significant enrichment was observed for the BPs “inflammatory response” (FE 1.95, 44 genes) and “cellular response to tumor necrosis factor” (TNF; FE 2.39, 15 genes). The former, inter alia, includes caspase 4, FAS receptor, NF-kB subunits (REL, RELA, RELB), and chemokine ligands (CXCL1, CXCL2, CXCL3, CXCL5, CXCL8). As to the TNF-related BP, the list of upregulated genes includes, e.g., the breast cancer type 1 susceptibility protein (BRCA1), RELA, interleukin-6 (IL6), and sirtuin 1 (SIRT1). The upregulation of genes coding for NF-kB subunits is consistent with the enrichment of the BPs “I-kappaB kinase/NF-kappaB signaling” (FE 3, 13 genes) and “NIK/NF-kappaB signaling” (FE 4.62, 5 genes). Additional upregulated genes listed in these two BPs were B-cell lymphoma 3 (BCL3), B-cell lymphoma 10 (BCL10), and NF-kB inhibitor like 1 (NFKBIL1). These results were corroborated by the significant enrichment of two KEGG pathways: “TNF signaling pathways” and “NF-kappa B signaling pathway” (FE 1.56, 31 genes, and FE 1.70, 23 genes, respectively).

Worthy of mention, several genes induced by AFB1 have a role in cell death, as demonstrated by the enrichment of BPs such as “negative regulation of apoptotic process” (FE 1.45, 52 genes) and “apoptotic processes” (FE 1.42, 50 genes).

Among the BPs most significantly enriched from AFB1-downregulated genes, we found some related to cell–cell interactions, i.e., “collagen fibril organization” (FE 3.3, 18 genes) and “extracellular matrix organization” (FE 2.25, 29 genes). This was confirmed by the enrichment of KEGG pathways like “ECM-receptor interaction” (FE 2.11, 30 genes) and “Regulation of Rho protein signal transduction” (FE: 2.10, 28 genes).

Additionally, AFB1 downregulated several genes involved in cholesterol synthesis as well as in steroid metabolism, thus resulting in the enrichment of the BPs “cholesterol biosynthetic process” (FE: 2.62, 13 genes) and “Steroid metabolic process” (FE: 3.53, 7 genes). Among the DEGs of the former BP, we found 24-dehydrocholesterol reductase (DHCR24), 7-dehydrocholesterol reductase (DHCR7), glucose-6-phosphate dehydrogenase (G6PD), apolipoprotein A1 (APOA1), and APOE. The steroid 5 alpha-reductase 1 (SRD5A1), sulfotransferase family 1A member 1 (SULT1A1), and SULT2B were among the DEGs represented by the BP “Steroid metabolic process”.

Finally, the BP “drug metabolism - cytochrome P450” was significantly enriched by AFB1 (FE 2.72, 24 genes); the main DEGs included GSTs (Table 5), flavin-containing monooxygenases 1, 2, and 5 (FMO2, FMO4, FMO5), the UDP glucuronosyltransferase 1 family, polypeptide A6 (UGT1A6), and the aldehyde dehydrogenase 3 family, member B1 (ALDH3B1). Some DEGs of this BP, i.e., CYP1A1, CYP1B1, and CYP2B6 (Table 4), are known to be also involved in carcinogenesis; as a consequence, a further BP enriched by AFB1 was “chemical carcinogenesis” (FE 2.39, 17 genes).

### 2.7. Targeted Gene Expression (qPCR)

To validate RNA-seq data, we measured the mRNA levels of a number of DEGs, known to be target of AFB1, by using targeted confirmatory qPCR assays (Figure S4). The obtained qPCR data for CYP3A28, SOD2, and GPX1 (upregulation), as well as for CYP1B1, NQO1, and NRF2 (downregulation), corroborated RNA-seq data. As to CYP1A1 and AHR, qPCR results were not entirely in agreement with those obtained by RNA-seq. Nevertheless, the trend of expression (upregulation for AHR and downregulation for CYP1A1) was conserved between the two technical approaches. Maybe the high standard deviation noticed between qPCR biological replicates prevented results to be statistically significant. Likewise, the high biological variability might be the reason why the mRNA expressions of CAT and SOD1, if assessed by means of RNA-seq, were not significantly downregulated by AFB1, but were significantly decreased if measured with the qPCR approach. As far as ARNT and KEAP1, in agreement with the RNA-seq data, no significant transcriptional variations were observed.

## 3. Discussion

For a better understanding of AFB1 mechanistic toxicology, whole-transcriptomic changes resulting from the exposure to this mycotoxin have been investigated in human hepatocytes, but scarce information are actually available for susceptible livestock species, except for turkeys and ducklings [6,43]. To the best of our knowledge, this is the first study evaluating the whole transcriptional profile of bovine liver cells exposed to AFB1, providing important information to explore bovine-specific traits characterizing the transcriptional response against this mycotoxin of worldwide concern.

### 3.1. Aflatoxin B1 Cytotoxicity

At first, we measured AFB1 cytotoxicity in BFH12 cells to rationally identify the best AFB1 concentration to be used in the consequent RNA-seq study. Notably, it might have been interesting to use a concentration of AFB1 physiologically relevant and found in bovine blood or body fluids. Nevertheless, this issue is quite challenging when talking about AFB1. As a matter of fact, cattle (likewise humans) may ingest feed contaminated with different amounts of AFB1; moreover, AFB1 exposure provokes a number of molecular, physiological, and biochemical effects in different organs and tissues, whose magnitude and consequences may differ substantially [55,56]. Therefore, selecting the best concentration of AFB1 to be used in in vitro experiments is sometimes difficult. Notably, the selected AFB1 concentration was in the range of those already used in similar whole-transcriptomic studies in humans and rodents [57,58].

Besides the known qualitative and quantitative differences between liver established cell lines and hepatocyte primary cultures (the gold standard for drug metabolism studies) [59], the BFH12 cell line is of fetal origin, hence presumably endowed with minor metabolic competence. To improve its responsiveness, we pre-treated cells with PCB126, a known AHR agonist [60]. Preliminary investigations showed the chosen PCB126 concentration (1 nM) did not induce cytotoxicity in BFH12 cells; moreover, it increased mRNA levels of some AHR gene battery members, thus supporting the usefulness of such an experimental approach.

The obtained AFB1 IC_50_ values were within the range of IC_50_ (1–16.9 μM) measured in previous studies made in HepG2 cells (a human hepatoma cell line) [61,62]. Interestingly, we also noticed that AFB1 cytotoxicity largely increased (lower IC_50_) when cells were pre-treated with PCB126, as recently demonstrated in human hepatocytes [63], thus suggesting the presence of additive/synergistic effects, possibly mediated via AHR receptors. Nevertheless, while PCB126 is a well-known AHR-ligand/agonist [60], this is not yet proven for AFB1 and, to a wider extent, aflatoxins [64].

### 3.2. Aflatoxin B1 Biotransformation

The obtained data demonstrated that a small amount of AFB1 was metabolized by BFH12 to produce AFM1 and AFL. The sum of AFB1, AFM1, and AFL detected in the medium was 1112 ng/mL, very close to the total amount of AFB1 administered to the cells (i.e., 3.6 μM; 1,124 ng/mL).

Moreover, the amount of AFM1, expressed as a percentage of AFB1, was similar to the one noticed in Madin–Darby canine kidney cells stably expressing the full-length bovine ABCG2 [65].

A controversial result is represented by the absence of AFM1 and AFL as well as the very low amount of AFB1 (i.e., close to 0 ng/mL) in cellular pellets, even though this latter confirms AFB1 absorption by BFH12 cells. In our experimental conditions, many cells died following AFB1 exposure, and we might hypothesize an increasing release of mycotoxin and its derivatives in the cellular medium. However, some additional evidence might be offered as explanation for such a behavior. The drug transporter ABCG2 reduces the in vitro uptake of AFB1 and mediates its secretion into milk, thus playing paradoxical protective and adverse roles with respect to AFB1 exposure [66]. In cattle, AFB1 and AFM1 are considered as likely ABCG2 substrates [67], and 6 μM AFB1 concentrations upregulated ABCG2 mRNA levels [68]. Finally, PCB126 significantly enhanced ABCG2 functional activity [67]. Overall, these facts might have contributed to the observed increasing efflux of AFB1, AFL, and AFM1 in the medium, though clear-cut evidence is not available. Worthy of note, characterization of AFB1 biotransformation was not the primary goal of our study, aiming to ascertain cytotoxicity and transcriptome changes triggered by AFB1 in the BFH12 cell line.

### 3.3. RNA-Seq Analysis of PCB126-Exposed Cells

When comparing cells exposed to PCB126 or DMSO, the RNA-seq data analysis showed that 1 nM PCB126 induced only small changes in the BFH12 transcriptome. These findings are consistent with those from a former study conducted in Hepa1c1c7 mouse hepatoma cells, in which only 119 DEGs were identified following a treatment with a 100 times higher PCB126 concentration [69]. Looking more carefully at the obtained results, an overexpression of CYP1A1 and CYP1B1 was expected and corroborated our qPCR preliminary results. Indeed, it is well-established that dioxin-like PCBs (DL-PCBs) bind and activate AHR [70,71], thereby inducing the members of CYP1A subfamily of drug metabolizing enzymes (i.e., CYP1A1, CYP1A2, and CYP1B1) [72,73].

Of potential interest is the upregulation of ABCB1, one important drug transporter limiting the bioavailability of xenobiotics by effluxing them from cells [74]; this would suggest this pump might play a role in PCB126 elimination. The role of ABCB1 (and its coded protein, i.e., the Permeability glycoprotein, P-gp) in PCBs efflux from bovine liver has never been investigated. However, an interaction between PCB126 and the human P-gp has been demonstrated in vitro [75]. Furthermore, in PCB-resistant killifish (*Fundulus heteroclitus*), exposure to PCB126 and PCB153 increased hepatic P-gp mRNA levels [76].

The upregulation of FOXQ1 seems to be consistent with previous studies suggesting an AHR-dependent increase in the expression of this transcription factor either in rat hepatic progenitor cells or in a rat hepatoma cell line exposed to PCB126 and TCDD, respectively [77]. Furthermore, FOXQ1 is a key player in hepatic carcinogenesis [78], and this would further corroborate PCB126 toxicological effects, as it is classified by the IARC as a group I carcinogen for humans [26].

### 3.4. RNA-Seq Analysis of AFB1-Exposed Cells

Whole-transcriptome analysis was performed by comparing cells exposed to AFB1 + PCB126 and those incubated only with PCB126. The bioinformatic analysis showed that AFB1 deeply affected the transcriptome of BFH12 cells. The total number of DEGs we obtained (6040, 22.7% of the entire cow genome) is consistent with previously published literature. For instance, incubation for 72 h of human liver HepaRG cells with 5 μM AFB1 showed a total of 4,603 DEGs (Benjamini–Hochberg adjusted *p* < 0.05, FC > 2) [40]; likewise, exposure of primary human hepatocytes for five days to a lower AFB1 concentration (0.3 μM, roughly corresponding to the IC_20_) resulted in 1,490 DEGs [79]. The DEGs resulting from AFB1 exposure were essentially associated with cancer, cellular damage and apoptosis, inflammation, and bioactivation and detoxification (CYPs and GSTs) pathways. These pathways are hereby more specifically discussed.

#### 3.4.1. Cancer

Differential expression analysis of the BFH12 transcriptome identified a large number of genes with known links to liver cancer in mammals, thus confirming the hepatocarcinogenic nature of AFB1 also in bovine liver cells.

Follistatin (FST) was one of the most significant genes upregulated by AFB1, which confirms what was previously reported in rat liver exposed to 1 ppm AFB1 in feed [80]. Imbalanced expression of follistatins in hepatoma cells is a well-known event [81], and it has been suggested that FST expression is required for proliferation and colony expansion of progenitor populations of hepatocytes [82].

Ornithine decarboxylase (ODC1) and A-Raf proto-oncogene as well as serine/threonine kinase (ARAF), both upregulated by AFB1, play a role in tumor promotion. Ornithine decarboxylase liver transcription increased in chicks exposed for 21 days to 2 mg/kg bw of AFB1 [4] as well as in rats given by gavage 50 μg/kg bw AFB1 [83]. The induction of ODC1 might contribute to increased cellular proliferation rates, which in turn predisposes to, and possibly potentiates, HCC. Moreover, ODC1 is overexpressed in several human cancers such as colon, liver [84], and breast carcinomas [85]. A similar role can be hypothesized for ARAF; indeed, an overexpression of RAF kinases (i.e., ARAF, BRAF, and CRAF) is associated with activation of the ERK pathway, which in turn increases cell proliferation in vitro and in vivo [86].

As regards the top genes that turned out to be downregulated by AFB1, the natriuretic peptide receptor 3 (NPR3) seems of particular interest for carcinogenesis, as its inhibition in renal cells has been shown to promote cancer development [87]. Overall, some mechanisms blocking cancer development appeared to be suppressed by AFB1. As an example, some collagen isoforms inhibiting cancer cell proliferation, i.e., Collagen type XVIII alpha 1 chain (COL18A1) and collagen type I alpha 2 chain (COL1A2), were downregulated. The former gene is a potent angiogenesis inhibitor, thereby affecting tumor growth. Notably, COL18A1 mRNA expression is lower in HCC patients compared to healthy controls [49]. The second collagen isoform coding gene (COL1A2) has been previously reported to inhibit colorectal cancer cell proliferation, migration, and invasion in a P53-dependent mechanism [50]. Additional genes with known tumor-suppressor activity were downregulated by AFB1. An interesting example is represented by the SWI/SNF-related, matrix-associated, actin-dependent regulator of chromatin, subfamily A, member 2 (SMARCA2); inactivating mutations or genetic alterations (e.g., epigenetics, copy number variants) have been frequently observed in HCCs [88] and particularly in alcohol-related HCC [89].

Looking at GSEA results and the significantly enriched GSs, some key cancer-related pathways were significantly overrepresented in AFB1-exposed cells compared to controls. These GSs included genes upregulated following KRAS activation (“KRAS signaling up”) as well as genes regulated by MYC (“MYC targets” v1 and v2). Interestingly, also the GS “KRAS signaling dn” was significantly enriched by downregulated DEGs. Members of RAS and MYC families of proteins are cooperating oncogenes, and their interplay and interdependency in driving cancer development and maintenance are well-established phenomena [90]. The most important member of RAS proteins is KRAS, which represents one of the most important drivers of cancer [91]. However, MYC is a nuclear oncogenic transcription factor playing a pivotal role in human carcinogenesis [92], being highly expressed in more than 80% of human cancers [93]. Notably, AFB1 chronic exposure has been previously shown to induce the expression of MYC in rat livers [94].

Turning our attention to protective mechanisms, the hallmark GS “P53 pathway” was enriched, with several P53-dependent genes resulting in being upregulated in AFB1-treated cells. It is well-established that AFB1 causes DNA damage [95], and the present induction of molecular networks linked to tumor suppressor P53 most likely aims to protect cells against the accumulation of deleterious mutations. Similarly, the activation of P53-regulated signaling pathways has been previously reported either in vivo in mice sub-chronically treated with AFB1 [96] or in vitro in human colorectal carcinoma cells treated for 24 h with 0.1, 1, 5, and 10 μM AFB1 [58].

#### 3.4.2. Cellular Damage—Apoptosis

The occurrence of DNA damage in BFH12 cells is suggested by the evidence, and significant enrichment of the hallmark GSs “DNA repair” and “G2M checkpoint” is the most evocative. After AFB1 exposure, cells increased mRNA levels of genes involved in DNA integrity checking and DNA repair mechanisms before entering mitosis; among these ones, we found a significant number of genes usually activated in response to ultraviolet radiation, an event that is well known to induce DNA damage (i.e., the GS “UV response up”). On the contrary, several genes whose expressions are typically reduced by UV radiation (i.e., the GS “UV response dn”) were downregulated by AFB1.

An alternative option to repair DNA defects consists in eliminating damaged cells by triggering apoptosis. Indeed, in the present experimental conditions the hallmark GS “Apoptosis” and the BPs “negative regulation of apoptotic process” and “apoptotic processes” were significantly enriched. As a whole, these results corroborate previous in vivo studies in which AFB1 triggers those mechanisms regulating hepatic programmed cell death via the FAS cell surface death receptor (FAS) [97,98]. Notably, in the present work FAS was significantly upregulated by AFB1. On the other hand, our transcriptional data also suggest AFB1 most likely increased the expression pattern of genes inhibiting apoptosis, thus blocking the death of damaged cells and possibly favoring tumorigenic processes. In this respect, a relevant gene is represented by the baculoviral IAP repeat containing 3 (BIRC3), a gene encoding for a member of the inhibitors of apoptosis family proteins, which regulate not only caspases and apoptosis but also inflammatory signaling and immunity, MAP kinase signaling, as well as cell proliferation, invasion, and metastasis. Recently, BIRC3 has been shown to induce HCC proliferation and metastasis in vitro and in vivo [99].

#### 3.4.3. Inflammation

Inflammatory response is a critical step in AFB1-induced liver injury [100]. The nuclear factor kappa B gene (NF-κB), as a key regulator of cellular stress in hepatocytes, controls inflammation, apoptosis, and cell injury [101]. A recent study performed in HepG2 cells demonstrated that treatment with the IC_50_ (i.e., 9 μM) of AFM1 for 48 h increased the amount of both IL6 and IL8 [25]. Likewise, IL6 mRNA levels were significantly induced in the liver of broilers exposed to AFB1 compared to controls [102]. Notably, upregulation of inflammatory cytokines represents an important factor in causing liver cancer [103], and IL6 is believed to play a key role in inflammation-associated tumorigenesis [104,105]. Our whole-transcriptomic analysis pointed out that AFB1 triggers inflammatory processes in bovine BFH12 cells. The outputs of GSEA and DAVID analyses highlighted significant enrichment of hallmark GSs, BPs, and KEGG pathways related to inflammation, and particularly of tumor necrosis factor (TNFα), and NF-kB signaling pathways. All members of the NF-κB gene family (NFKB1, NFKB2, RELA, RELB, REL) were induced by AFB1, with NFKB2 showing a particularly high FC (i.e., 7.2). It is conceivable to hypothesize that activation of the NF-κB pathway in BFH12 cells treated with AFB1 is mediated by TLR2, whose expression was 8.44 times higher than in controls. As a result of NF-κB signaling pathway activation, several genes encoding for inflammatory cytokines were induced, including IL6 and IL8. Moreover, also the mRNA levels of interleukin-1A (IL1A), a potent pro-inflammatory cytokine, were greatly upregulated by AFB1 (FC = 80), thus confirming what was previously observed in the liver of rats injected with AFB1 [103]. Nonetheless, in liver, NF-κB signaling is triggered by IL1A produced by dying hepatocytes [106,107]. As far as TNFα is concerned, we did not observe significant changes in gene expression. However, several genes known to be regulated by this inflammatory cytokine, such as activating transcription factor 3 (ATF3) and TNFα-induced proteins (e.g., TNFAIP1, TNFAIP2, TNFAIP3), were upregulated by AFB1.

Among the AFB1-downregulated DEGs, significant enrichment of the hallmark GSs “estrogen response early” and “estrogen response late” is of particular interest, if considering a possible cross-talk between HCC and estrogens. Despite several aspects still remaining indecipherable, estrogens have been shown to possess anti-HCC activity, and such a protective/counteracting role might be linked to their anti-inflammation effect [108]. In particular, estrogens inhibit IL6 production, thus attenuating the downstream inflammatory processes influencing hepatocarcinogenesis [108]. The results we obtained in BFH12 cells exposed to AFB1 would go in this direction; an overall downregulation of genes known to be controlled by estrogens was observed, resulting in a reduced cellular response to estrogenic signals, possibly favoring the rise of inflammatory processes.

#### 3.4.4. Bioactivation, Detoxification, and Transport across Cell Membranes: CYPs and GSTs and ATP Binding Cassette (ABC) Efflux Transporters

Considering the overall importance of CYPs in xenobiotic metabolism, including that of AFB1, we carefully assessed transcriptional data related to this superfamily of drug metabolizing enzymes. In BFH12 cells, the mRNA levels of CYP1A1 and CYP3A28 were significantly modulated by AFB1; on the contrary, CYP1A2, playing an important role in AFB1 bioactivation to AFBO in humans and turkeys [109], was not constitutively expressed in this cell line; hence, its potential contribution to AFB1 biotransformation (and toxicity) remains to be clarified. In our experimental conditions, CYP3A28 gene expression was greatly induced by AFB1 (FC = 16). Present data would corroborate results previously obtained in vitro in the human HepG2 cell line (10 μM, 24 h) and hepatocyte primary cultures (increasing concentrations ranging from 0.001 to 50 µM, 24 h), in which AFB1 activated the pregnane X receptor (PXR, an important liver transcription factor); this latter, in turn, increased CYP3A4 gene transcription [57,110]. However, the human CYP3A4 orthologue of domesticated turkeys (i.e., CYP3A37) is inhibited by AFB1 [44].

Concerning CYP1A1, AFB1 slightly decreased its mRNA levels (FC = 2.43). Mycotoxin was shown to downregulate this gene in chicken liver [111] as well as in rabbit hepatocyte primary cultures exposed to 0.1 and 1 μM AFB1 for 72 h [112]. Nevertheless, the current scientific literature suggests to us the presence of different scenarios: AFB1 can upregulate CYP1A1 gene expression, as shown in human hepatocytes [110] and a rat hepatoma cell line (5 μM AFB1, 0.5, 2, 4, 8, 16 h) [113]; but, at the same time, it may result in any transcriptional change [42].

The CYP1 family of drug metabolizing enzymes is composed of three members: CYP1A1, CYP1A2, and CYP1B1 [114]. In BFH12 cells pre-treated with PCB126, exposure to AFB1 did not result in possible alteration of CYP1B1 mRNA levels; on the contrary, PCB126 alone upregulated CYP1B1 gene expression when compared to DMSO (control). In vitro, the human heterologously expressed (human lymphoblastoid cell line) CYP1B1 has appeared to be particularly efficient at activating AFB1 [115]; moreover, CYP1B1 has been reported to be regulated by AFB1 (32 and 320 μM, 2 h) only in human monocytes [116]. Therefore, the hypothesis that AFB1 and PCB126 may be substrates and, consequently, show agonistic/antagonistic effects on CYP1B1 cannot be excluded, albeit confirmatory studies are clearly needed.

In a wider scenario, it is conceivable to suppose that pre-treatment with PCB126, a known AHR ligand and CYP1A1 inducer, might have represented a confounding factor in interpreting and comparing present CYP1 family data with those available in the literature and mostly relating to AFB1 alone. Regarding this, 1 μM PCB126 caused an increase in CYP1A1 (17-fold compared to vehicle) and CYP1B1 (75-fold) gene expression in human umbilical vein endothelial cells (HUVECs) [117]. Nevertheless, it must be emphasized how the PCB126 concentration here used (1 nM, definitely 1000× lower) identified only eight DEGs with higher mRNA levels compared to DMSO, albeit CYP1A1 and CYP1B1 were among these ones. Furthermore, the whole-transcriptomic analysis of cells exposed to AFB1 + PCB126 was made by comparison to those incubated merely with PCB126. Hence, the PCB126 “background”, “noise effect”, or “interfering role” should have been deleted. Obviously, this is the result of potential cross-talk, whose presence and biological impact (both molecules might at the same time be present in the living organism) need to be investigated more in depth.

Additional CYPs were significantly modulated by AFB1. Most of these CYP isoforms are primarily involved in the metabolism of endogenous molecules rather than xenobiotics and mycotoxins. For instance, CYP26B1 (upregulated by AFB1) is a retinoic acid hydroxylase that metabolizes retinoic acid, a vitamin A-derived biomolecule essential for cell growth, differentiation, and embryonic development. Likewise, CYP27B1 (upregulated) is the enzyme converting vitamin D in its active form, and its overexpression has been shown to inhibit growth in human hepatocytes, thus exerting an anti-HCC activity [118]. Finally, aromatase (CYP19A1) appeared to be significantly downregulated by AFB1. This is in opposition with what was previously reported in human choriocarcinoma trophoblastic cells and an MCF-7 cell line treated with 10 μM (1 h) and 1 μM (72 and 96 h) AFB1, respectively [119,120]. Such a discrepancy might be attributed to the different in vitro model we used or to species differences. However, this result is in agreement with the overall downregulation of several genes known to be controlled by estrogens (see GSEA data).

A second family of drug metabolizing enzymes playing a major role in AFB1 detoxification is GSTs. In humans, GSTM1, GSTT1, and GSTM2 are the enzyme isoforms mostly involved in AFBO conjugation with glutathione, whereas GSTP1, GSTA1, and GSTA2 have a minor role in AFB1 detoxification [109]. In the present study, two putative GSTs, namely GSTT1 and GSTA2, were downregulated by AFB1; on the contrary, GSTP1 and GSTO1 mRNA levels were significantly increased, albeit with low fold changes. As shown in a previous study investigating the whole-transcriptomic effects of AFB1 on turkey liver, the different GST isoforms seemed to respond differently to the mycotoxin (i.e., induction, inhibition, no effect) [44]. As an example, transcriptomic investigations made in ducklings showed that hepatic GST1, GST3, and GSTK1 mRNA levels were increased by AFB1 [43], whereas in rats only the GSTA5 gene was upregulated [121].

Overall, most hepatic GST isoforms constitutively expressed in BFH12 (10 out of 13) were downregulated by AFB1. Similar evidence was previously reported in the livers of chicks fed AFB1 for 21 days [4]. The GST pattern of expression has been considered as a predictor of AFB1 resistance [122]; if so, the overall inhibition we observed in BFH12 cells might be indicative of an inability of this in vitro model to cope with AFB1-induced toxicological effects; however, this hypothesis needs to be confirmed by further molecular studies.

Finally, two important efflux transporters, namely the ATP binding cassette subfamily G members 1 and 2 (ABCG1 and ABCG2), were inhibited by AFB1, with FCs of 64 and 2.4, respectively. A relationship between AFB1 exposure and ABCG1 gene expression has never been observed in the literature. This efflux transporter is a cholesterol lipid efflux pump that plays a well-known role in tumor growth, conferring chemoresistance (e.g., vs platinum-based anticancer drugs) to various malignant tumors, including HCC [123,124]. Nevertheless, the great FC we observed made us speculate a possible pivotal role of this gene in response to AFB1 exposure, thus requiring a better understand of the role of ABCG1 in AFB1 mechanistic toxicology.

On the contrary, a number of in vitro studies proved the interaction between AFB1 and murine, human, and bovine ABCG2; overall, this evidence leads one to conclude AFB1 could be a substrate of this important efflux pump [66,67]. However, in our experimental conditions, neither AFB1 nor its main derivatives (AFL, AFM1) were detected in the BFH12 cellular pellet; therefore, ABCG2 did not seem to play a crucial role in determining the AFB1 fate in cattle liver, more specifically in modulating its bioavailability and elimination. However, we cannot forget that a reduced expression of ABCG2 might greatly affect the bioavailability of other xenobiotics (including drugs) that are present in the liver and known to be substrates/actively transported outside the cell by this efflux transporter. As a consequence, the dual roles played by ABCG1/ABCG2 in AFB1 mechanistic toxicology require further confirmatory molecular studies.

#### 3.4.5. Comparison with Data from Human In Vitro Models

Overall, the whole-transcriptome profiles of BFH12 treated with 3.6 μM AFB1 showed some similarities with the results previously obtained on human hepatocytes. However, it is important to highlight that such comparison is a complex analysis that usually comes with a great variance and a limited overlap of DEGs [125]. This comes from the fact that (i) we are comparing cells isolated from different species (i.e., bovine and human), and a number of human cell lines (i.e., HepaRG, HepG2, primary hepatocytes) have been used; (ii) cell incubation conditions (e.g., AFB1 concentration and exposure duration) are considerably different; and (iii) omics platform and data analysis can be extremely diverse (e.g., microarray or RNA-sequencing). For example, if looking at the 30 most frequent and strongest DEGs affected by AFB1 in HepaRG cells (reported in [125]), only 10 genes were identified as DEGs in the present study, thus highlighting a certain difference between bovine and human in vitro models.

For these reasons, we think that a general comparison between the present and the previously published data should be performed by focusing on the functional analysis outcomes, rather than DEGs. For instance, enrichment of the P53 and DNA damage pathways herein reported is in large agreement with previous studies performed in human cell lines. In particular, the P53 pathway was significantly enriched in HepaRG cells exposed to 1 μM AFB1 for 24 h [126], in induced pluripotent stem-cell (iPSC)-derived human hepatocytes incubated with 0.2 μM AFB1 for 7 days [127], and in HepG2 and human primary hepatocytes (HPH) treated with 1 μM and 1.25/5 μM for 24 h, respectively [41]. Thus, we might hypothesize that activation of the P53 pathway also represents a key point in bovine response to AFB1. Likewise, apoptosis seems to be a hallmark of AFB1 exposure in both human and bovine cells. In accordance to our results, gene sets and GO terms related to programmed cell death appeared to be significantly enriched in human HepG2, PHH, and HepaRG cell lines [41,126].

Looking at the pathways and the GO terms related to drug metabolism, the mRNA levels of several phase I/II drug metabolizers were affected by AFB1 (as discussed above). Indeed, drug metabolism is triggered also in human cells exposed to AFB1. In particular, biotransformation was significantly enriched in HepG2, HepaRG, and PHH [40,41].

Finally, enrichment of the BP “inflammatory response” and the “NF-kappa B signaling pathway” is interesting evidence that highlights a distinctive transcriptional signature of the bovine cellular model here used. Indeed, in previous studies assessing the effects of AFB1 in human hepatic cells, these GO terms did not appear significantly enriched [40,41,126,127]. It is conceivable to hypothesize AFB1 modulates the expression of several genes implicated in cattle inflammatory processes (e.g., chemokine ligands, TNFα and INFγ pathways), which in part are modulated by the pivotal transcription factor NF-kB.

Overall, this evidence suggests bovine liver cells respond to AFB1 through molecular mechanisms occurring also in human models, but triggering also a specific set of target genes typically involved in the inflammatory process.

## 4. Conclusions

To the best of our knowledge, this is the first study assessing the overall in vitro transcriptional effects of AFB1 in cattle; in particular, on an established fetal hepatocyte cell line (BFH12) and by using RNA-seq technology. From our experimental results and our review of the existing literature, we propose the following conclusions. (1) Aflatoxin B1 deeply affected the cattle transcriptome, and the number of DEGs (2,632 and 3,408 genes up- and downregulated, respectively) is consistent with those previously published with human hepatocytes. (2) The pathways mostly affected by AFB1 exposure are essentially cancer, cellular damage and apoptosis, inflammation, and drug metabolism and transport. The main up- or downregulated DEGs showed behavioral analogies with human gene orthologues; interestingly, some of these ones are involved in HCC carcinogenicity. (4) Nevertheless, the possible presence of species- and cell line-dependent differences in the constitutive expression, gene regulation, and transcriptional response to AFB1 cannot be excluded. As a matter of fact, BFH12 is a fetal hepatocyte cell line, showing a lower metabolic capacity compared to human hepatoma cell lines. This is the reason why we pre-treated BFH12 cells with low amounts of PCB126, a known AHR and CYP1A inducer. This might be considered a limitation of the study, but transcriptome data analysis took due consideration of this. (5) Prospective molecular studies are clearly needed to confirm the role played by specific pathways/genes (e.g., CYP1A, and 3A28; ABCB1, G1 and G2) in AFB1 mechanistic toxicology and, consequently, to evaluate the possible protective roles of natural compounds (e.g., antioxidants), low-cost adsorbents (e.g., clays), and probiotics (e.g., *Lactobacillus* spp.).

## 5. Materials and Methods

### 5.1. Materials

Cell culture flasks and multi-well plates were purchased from Sarstedt (Verona, Italy). Williams’ Medium E, l-alanyl-l-glutamine, penicillin/streptomycin, and standardized fetal bovine serum (FBS) were acquired from Biochrom (Biospa, Milan, Italy).

Aflatoxin B1 (from *Aspergillus flavus*; CAS Number 1162-65-8), dimethyl sulfoxide (DMSO), dexamethasone, insulin from bovine pancreas, and trypan blue were obtained from Sigma-Aldrich (St. Louis, MO, USA). The polychlorinated biphenyl AHR agonist 3,3′,4,4′,5-pentachlorobiphenyl (PCB126, 99%, CAS Number 57465-28-8) and AFM1 were purchased from Lab Service Analytica (Bologna, Italy). Aflatoxicol and ^13^C_17_-AFB1 were obtained from DBA Italia (Milano, Italy) and Orsell (Modena, Italy), respectively. All other chemicals used in the study were commercially available and of molecular biology grade. All solvents used for quantification of AFB1 metabolites were of LC-MS grade.

### 5.2. Cell Culture

The bovine SV40 large T-antigen-transduced fetal hepatocyte-derived cell line BFH12 was kindly provided by Dr. Axel Schoeniger (Institute of Biochemistry, University of Leipzig, Germany). Cells were cultured in Williams’ E medium containing 5% heat-inactivated FBS, 1% penicillin/streptomycin, 2 mM L-alanyl-L-glutamine, 100 nM dexamethasone, and 0.2 U/mL insulin at 37 °C and 5% CO_2_ in a humidified atmosphere, as previously described [46]. Medium was changed every 3 days, and cells were harvested every 7 days using Trypsin-EDTA (8 min at 37 °C). Cell number and viability were checked using the trypan blue dye exclusion test. For all the experiments, cells were used from passage 16 to passage 20 maximum. Furthermore, cell cultures were checked for *Mycoplasma* spp. contamination using the PCR Mycoplasma Test Kit (PromoKine, Heidelberg, Germany).

### 5.3. Preliminary Evaluations of BFH12 Responsiveness to PCB126

In established hepatic cell lines, the basal gene expression profiles, and the corresponding phenotype, may differ substantially from hepatocyte primary cultures, the gold standard for studying xenobiotics metabolism and toxicity [59]. As for the BFH12 cell line, a partial characterization of constitutive and inducible mRNA levels of foremost drug metabolizing enzymes and transporters has already been made [45,46]; however, no whole-transcriptomic data have been published so far. Assuming the metabolic competence of fetal hepatocytes is lower compared to that of adult liver cells, we opted for pre-treatment with an AHR agonist, i.e., PCB126, to increase the cell line metabolic competence, hence the responsiveness to AFB1. Nonetheless, the combined use of PCB126 and AFB1 is not novel [63,67]. No information on the BFH12 transcriptomic changes resulting from exposure to PCB126 was available in the literature; hence, a first set of preliminary experiments was undertaken to define the best PCB126 incubation protocol to boost the cellular whole-transcriptomic response to AFB1.

Briefly, after a preliminary evaluation of cellular viability exposing BFH12 cells to increasing concentrations of PCB126 (1, 10, and 100 nM) for 24 h (for details see paragraph 5.4), BFH12 cells (5 × 10^4^ cells/well, 6-well culture plates) were exposed to the same PCB126 concentrations for 6, 12, and 24 h; at the end of the incubation step, monolayers were scraped off, and total RNA was used to measure the time- and concentration-dependent AHR gene battery upregulation by using well-established qPCR protocols (see Section 5.9 for details).

### 5.4. Aflatoxin B1 Cytotoxicity

Cells were seeded in 96-well flat-bottom plates (Sarstedt) at a density of 6 × 10^3^ cells/well. Once reaching confluency (4 days after seeding), cells were exposed to increasing concentrations of AFB1 (range 0.04–43.3 μM) for 24, 48, and 72 h. AFB1 was dissolved in DMSO (v:v), whose final concentration never exceeded 0.1%. All incubations with the mycotoxin were performed using media without FBS to avoid possible binding of a considerable proportion of AFB1 to serum albumin [128]. Moreover, in each experiment, cells treated with the vehicle only or untreated were included as controls.

Cell Proliferation Reagent WST-1 (Roche, Basel, Switzerland) was used to measure cell viability. This assay is based on the fact that NADH, produced in the mitochondrial of living cells, reduces WST-1, a tetrazolium salt, into formazan that can be measured via colorimetry. At the end of each incubation, 5 μL of WST-1 reagent was added to each well. After 3 h of incubation (37 °C, 5% CO_2_), the absorbance was measured at 450 and 690 nm (reference wavelength) by using a VICTOR™X4 Multilabel Plate Reader (Perkin Elmer, Waltham, MA, USA). The cell viability was expressed as the percentage relative to that of cells exposed to the vehicle only (0.1% DMSO). Experiments were performed in triplicate, and each concentration was tested in sextuplicate.

### 5.5. Cell Incubation for Gene Expression Analyses

**Figure 3:**
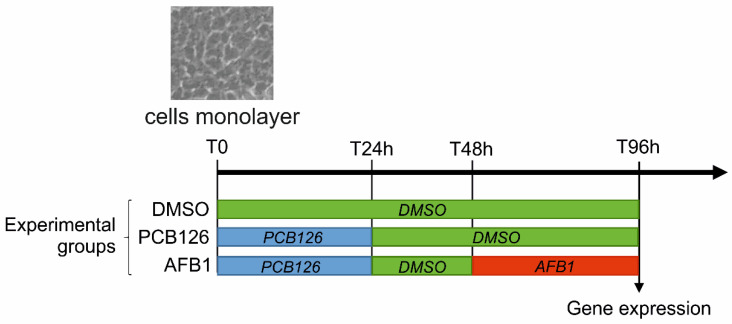
Scheme reporting the cell treatments performed in this study and the resulting experimental groups.

To measure the effects of AFB1 on the whole BFH12 transcriptome (RNA-seq and confirmatory qPCR), cells were seeded in 6-well culture plates at a density of 5 × 10^4^ cells/well. Four days after (T0), exhausted medium was changed and cells were incubated with 1 nM PCB126. After 24 h of treatment (T24h), PCB126 treatment was stopped, the medium was changed, and cells were further incubated with DMSO for 24 h. Forty hours from the onset of the experiment (T48h), medium was changed again, and monolayers were exposed to 3.6 μM AFB1 for further 48 h, for an overall length of the experiment of 96 h (T96h; see Figure 3).

Polychlorinated biphenyl-126 and AFB1 concentrations were chosen based on results obtained with either the abovementioned preliminary studies (PCB126: see paragraph 5.3) or cytotoxicity (AFB1). Notably, the selected AFB1 concentration was in the range of concentrations commonly tested in previous studies involving human hepatic cell lines [57,58]. Cells treated with 0.1% DMSO were used as control. At the end of the incubation time, the medium was removed, cells were washed with PBS/EDTA 1X, scraped off, and centrifuged at 4 °C for 5 min at a speed of 14,000 rpm. Supernatant was removed, and cell pellets were re-suspended in 600 μL of RLT buffer (Qiagen, Hilden, Germany) containing 6 μL of β-mercaptoethanol. In order to disrupt pellets, samples were vortexed and stored at −80 °C until use. Total RNA was extracted with the RNeasy Mini kit (Qiagen), following the manufacturer’s instructions, and quantified by using the NanoDrop 1000 Spectrophotometer (Thermo Fisher Scientific, Waltham, MA, USA). Total RNA quality was assessed with a 2100 Bioanalyzer (Agilent Technologies, Santa Clara, CA, USA). All samples had an RNA Integrity Number (RIN) value > 7.

### 5.6. RNA-Seq Library Preparation and Sequencing

For each experimental condition (i.e., DMSO, PCB126, AFB1), three independent biological replicates (i.e., independent cell culture experiments) were considered. As a consequence, a total of nine tagged RNA-seq libraries were prepared using Agilent’s SureSelect Strand Specific RNA Library Preparation Kit (Agilent Technologies) following the manufacturer’s instruction. Briefly, poly(A) mRNA was purified from 400 ng of total extracted RNA and fragmented using an RNA-seq Fragmentation Mix. First-strand and second-strand cDNA were synthetized and end-repaired. Adenylation of cDNA 3′ ends and adaptor ligation were performed. Twelve cycles of PCR were used to amplify and index the adaptor-ligated cDNA library. The PCR products were then purified, size-selected using the SPRIselect reagent kit (Beckman Coulter, Brea, CA, USA), and eluted in 21 μL of RNAse-free water. Library concentrations were measured using either a Qubit RNA Assay kit (Life Technologies, Carlsbad, CA, USA), in a Qubit 2.0 Fluorometer (Life Technologies), or a PCR-based method using the NEBNext Library Quant Kit for Illumina (New England Biolabs, Ipswich, MA, USA). Individual libraries were monitored for insert size using the Agilent DNA 1000 assay kit (Agilent Technologies) on the Agilent Bioanalyzer 2100 instrument (Agilent Technologies). Multiplexed single-end sequencing (50 bp) was carried out on an Illumina Hi-Seq 4000 (Fasteris, Geneva, Switzerland).

### 5.7. Differential Expression Analysis

Initial quality control was carried out using the FastQC software, version 0.11.9 [129]. Reads were trimmed, and adapters were removed using Trimmomatic (version 0.36), with default parameters [130]. Reads shorter than 36 bps were excluded. To filter out any remaining post-sequencing ribosomal RNAs (rRNAs), the local sequence alignment tool SortMeRna 2.0 [131] was applied against different databases (Rfam 5.8S; Rfam 5S; Silva 16S archaeal, bacterial; Silva 18S eukaryote; Silva 23S archaeal, bacterial; Silva 28S eukaryote). Reads were then mapped against the reference *Bos taurus* genome, available in NCBI (ARS-UCD1.2), using the STAR aligner [132] and following the two-pass mapping mode. The maximum number of allowed mismatches and the maximum number of loci the read was allowed to map to were set to 4 and 10, respectively. Read counts for each sample, at the gene level, were extracted by setting the GeneCounts quantification while running STAR. Extracted read counts were used for the analysis of differential gene expression and were conducted in EdgeR [133]. Samples were grouped according to treatments (i.e., DMSO, PCB126, AFB1), and pair-wise analyses were performed to assess the transcriptional changes induced by PCB126 (i.e., PCB126 vs DMSO) and AFB1 (i.e., AFB1 vs PCB126). Genes showing an extremely low expression level in all samples were filtered out by using the filterByExpr function, and extracted reads were normalized with the trimmed mean of M-values (TMM) method. After estimating common and tag-wise dispersions (estimateDisp), and fitting a linear model (glmQLFit), the function glmTREAT was used to find out the differential expressed genes (DEGs), with FDR set at ≤0.05 and a threshold for a significant Fold Change (FC) set at ≥1.5. The complete R code used for the differential expression analysis is reported in File S1.

### 5.8. Enrichment Analysis

A functional interpretation of DEGs was obtained through an enrichment analysis made using the Database for Annotation, Visualization, and Integrated Discovery (DAVID) software v. 6.8 [134]. ‘‘Biological processes’’ (BP) and “KEGG Pathways” were used by setting the “gene count” equal to 3 and the “ease” value equal to 0.01. Bovine Ensembl gene identifiers were used to establish in DAVID two different gene lists (i.e., significantly up- and downregulated genes) and a “background” (i.e., all the expressed genes).

A pre-ranked Gene Set Enrichment Analysis (GSEA) [135] was performed to determine whether gene sets defined a priori showed statistically significant enrichment at either end of the ranking. A statistically significant enrichment value (nominal *p* value < 0.05) indicated that the biological activity (e.g., the biomolecular pathway), characterized by the gene set, was correlated with the supplied ranking. As GSEA recognizes gene names instead of Ensembl Gene IDs, these were obtained in Ensembl BioMart using, as an input, the Ensembl Gene IDs. The input was prepared as follows: the raw *p* values obtained through the DE analysis (i.e., EdgeR, AFB1 vs PCB126) were used to rank the list of genes by significance. When multiple genes with the same gene name were detected, only the most significant one (based on *p* value) was retained. To specify the direction of the gene expression variation, the *p* values (pval) were replaced by 1-pval or -(1-pval) when a gene was over-expressed or under-expressed in the “AFB1” group, respectively. The analysis was carried out by using a classic enrichment statistics and Molecular Signatures Database (MSigDB) curated gene sets (i.e., Hallmarks). A nominal *p*-value of 5% was used as threshold of significance.

### 5.9. Quantitative Real-Time PCR

The same RNA used for the RNA-seq experiments was used for confirmatory qPCR analyses. Overall, complementary DNA (1 μg) was synthetized using the High-Capacity cDNA Reverse Transcription kit (Applied Biosystems, Foster City, CA, USA). qPCR amplification was carried out in a final volume of 10 μL, using 2.5 μL of cDNA, the Power SYBR Green PCR Master Mix (Applied Biosystems), and the Stratagene M × 3000P thermal cycler (Agilent Technologies). The whole list of target genes and primers used for qPCR analyses are reported in Table S4. The quality of each qPCR assay was gathered from standard curve slopes and correlation coefficients. The PCR efficiency (E) was measured by using the equation Ex = 10^−1^/slope, and only *E* values between −3.6 and −3.1 were considered acceptable (Table S4). The Ribosomal Protein Lateral Stalk Subunit P0 (RPLP0) and Tata Binding Protein (TBP) were used as reference genes, as their expression did not show statistically significant differences between control and treated groups. Therefore, the arithmetic mean of *Ct* values reported for RPLP0 and TBP was used for sample normalization. Messenger RNA relative quantification (RQ) was performed by using the ΔΔCt method [136]. The calibrator sample consisted of cDNA obtained from a bovine liver. Data are expressed as fold change of treated versus untreated cells (DMSO) ±mean standard error (SEM).

### 5.10. Analytical Investigations

Total AFB1, AFM1, and AFL were measured in both medium and cells exposed to AFB1 3.6 μM for 48 h (Figure 3). After being thawed at room temperature, cells (resuspended in 200 µL of ultrapure water) and medium samples were vortexed for 15 s and centrifuged at 12,000× *g* for 5 min. A 20 µL aliquot of the supernatant was transferred into a vial containing 120 µL of water:acetonitrile 80:20 (*v*/*v*), with 0.1% formic acid and 10 µL of internal standard solution (200 ng/mL ^13^C_17_-AFB1 in acetonitrile). Samples were then vortexed for 30 s and injected onto a LC-MS/MS system consisting of a Waters Acquity UPLC binary pump coupled to a Quattro Premier XE triple quadrupole mass spectrometer (Waters, Milford, MA, USA). Chromatographic separation was obtained using a Waters Acquity BEH C18 (50 × 2.1 mm, 1.7 µm) reversed-phase column (Waters) during a 5 min run under programmed conditions, with mobile phase consisting of a mixture of acetonitrile and 0.1% formic acid in water, at a flow rate of 0.3 mL/min. The instrument was equipped with an electrospray ionization source (ESI) operating in positive mode at a capillary voltage of 3.75 kV, with source and desolvation temperatures set at 120 and 350 °C, respectively. Desolvation gas flow was 650 L/h and cone gas flow was 100 L/h. The specific transitions monitored for each analyte were: 313.1 > 284.7 m/z (CV 53 V; CE 25 eV) for AFB1, 329.1 > 272.7 m/z (CV 42 V; CE 26 eV) for AFM1, 297.1 > 268.4 m/z (CV 48 V; CE 20 eV) for AFL, and 330.3 > 300.6 m/z (CV 50 V; CE 22 eV) for the internal standard 13C17-AFB1. Data were acquired and processed with Waters MassLynx software (version 4.1, Milford, MA, USA).

### 5.11. Statistical Analysis

Dose–response curves were obtained by using GraphPad Prism software (version 8.0.2, San Diego, CA, USA). Mortality rates of the treated cells compared to control cells (DMSO) were reported, and a non-linear regression (log(inhibitor) vs. normalized response, variable slope) was built. The half-maximal inhibitory concentration (IC_50_) and the goodness-of-fit (R squared) were provided by the software.

Statistical analysis of qPCR data, aiming to confirm mRNA changes induced by AFB1 and measured by RNA-seq, was performed by using the nonparametric Mann–Whitney U-test, with the level of significance set at *p* ≤ 0.05.

## Figures and Tables

**Table 1:** Biotransformation of AFB1 in PCB126-preincubated BFH12 cells. Concentrations of AFB1, AFM1, and aflatoxicol (AFL) were measured in the medium and in cellular pellets after 48 h of exposure to 3.6 μM AFB1 (i.e., at the end of the experiment, T96 h). Data are expressed as mean concentration ± standard deviation of five independent cell culture experiments.

	AFB1 (ng/mL)	AFM1 (ng/mL)	AFL (ng/mL)
Medium	974.24 ± 186.09	45.6 ± 3.78	92.8 ± 10.50
Cellular pellet	1.43 ± 0.99	0	0

**Table 2:** List of genes upregulated after cell pre-treatment with PCB126. For each differentially expressed gene (DEG), the Ensembl gene ID, log_2_ fold change (logFC), and False Discovery Rate (FDR) are provided as reported in edgeR output. The Ensembl gene description and acronym are also provided. NA = not available.

Ensemble Gene ID	logFC	FDR	Gene Description	Gene Acronym
ENSBTAG00000001021	4.00	4.61 × 10^−5^	Cytochrome P450 family 1 subfamily A member 1	CYP1A1
ENSBTAG00000010531	1.79	4.93 × 10^−5^	Cytochrome P450, family 1, subfamily B, polypeptide 1	CYP1B1
ENSBTAG00000026527	4.56	0.00014	NA	NA
ENSBTAG00000005997	0.97	0.00611	Multidrug resistance protein 1	ABCB1
ENSBTAG00000052132	1.84	0.00618	Forkhead box Q1	FOXQ1
ENSBTAG00000048991	1.18	0.00618	REC114 meiotic recombination protein	REC114
ENSBTAG00000000494	1.29	0.01107	Phosphodiesterase 4D	PDE4D
ENSBTAG00000017466	1.29	0.01110	Hyperpolarization activated cyclic nucleotide gated potassium channel 4	HCN4

**Table 3:** Top-ten genes up- (↑) and downregulated (↓) by AFB1 exposure. For each DEG (AFB1 vs PCB126), Ensembl gene ID, log_2_ fold change (logFC), and False Discovery Rate (FDR) are provided as reported in edgeR output. The Ensembl gene description and acronym are also provided.

Ensembl Gene ID	logFC	FDR	Gene Description	Gene Acronym	Pattern of Expression
ENSBTAG00000017425	6.38	1.74 × 10^−17^	Gap junction protein beta 2	GJB2	**↑**
ENSBTAG00000003329	3.40	1.98 × 10^−16^	Follistatin	FST	**↑**
ENSBTAG00000011316	4.09	7.33 × 10^−16^	Zinc finger CCCH-type containing 12A	ZC3H12A	**↑**
ENSBTAG00000004256	2.91	1.06 × 10^−15^	Ornithine decarboxylase 1	ODC1	**↑**
ENSBTAG00000005039	2.65	1.24 × 10^−15^	A-Raf proto-oncogene, serine/threonine kinase	ARAF	**↑**
ENSBTAG00000019716	7.54	1.24 × 10^−15^	C-X-C motif chemokine ligand 8	CXCL8	**↑**
ENSBTAG00000014324	2.97	1.50 × 10^−15^	ANTXR cell adhesion molecule 2	ANTXR2	**↑**
ENSBTAG00000011358	3.74	1.63 × 10^−15^	Immediate early response 3	IER3	**↑**
ENSBTAG00000000507	3.62	2.80 × 10^−15^	Nuclear receptor subfamily 4 group A member 1	NR4A1	**↑**
ENSBTAG00000001864	5.47	3.68 × 10^−15^	Nuclear receptor subfamily 4 group A member 3	NR4A3	**↑**
ENSBTAG00000007665	−3.35	1.98 × 10^−16^	Natriuretic peptide receptor 3	NPR3	**↓**
ENSBTAG00000013848	−4.20	1.98 × 10^−16^	Adhesion G protein-coupled receptor D1	ADGRD1	**↓**
ENSBTAG00000023907	−3.52	1.98 × 10^−16^	Collagen type XVIII alpha 1 chain	COL18A1	**↓**
ENSBTAG00000004231	−5.70	1.98 × 10^−16^	Glycoprotein M6A	GPM6A	**↓**
ENSBTAG00000007494	−3.34	1.98 × 10^−16^	SWI/SNF related, matrix associated, actin dependent regulator of chromatin, subfamily a, member 2	SMARCA2	**↓**
ENSBTAG00000013391	−3.66	2.19 × 10^−16^	ANKH inorganic pyrophosphate transport regulator	ANKH	**↓**
ENSBTAG00000013472	−3.83	2.24 × 10^−16^	Collagen type I alpha 2 chain	COL1A2	**↓**
ENSBTAG00000034693	−5.91	2.79 × 10^−16^	Synaptotagmin 1	SYT1	**↓**
ENSBTAG00000001405	−3.78	2.99 × 10^−16^	Glucuronic acid epimerase	GLCE	**↓**
ENSBTAG00000017448	−4.08	3.46 × 10^−16^	EGF containing fibulin extracellular matrix protein 1	EFEMP1	**↓**

**Table 4:** Gene expression (↑, upregulation; ↓, downregulation) of CYP isoforms after AFB1 treatment. For each DEG (AFB1 vs PCB126), Ensembl gene ID, log_2_ fold change (logFC), and False Discovery Rate (FDR) are provided as reported in edgeR output. The Ensembl gene description and acronym are also provided.

Ensembl Gene ID	logFC	FDR	Gene Description	Gene Acronym	Pattern of Expression
ENSBTAG00000012212	5.16	1.3 × 10^−14^	cytochrome P450, family 26, subfamily B, polypeptide	CYP26B1	**↑**
ENSBTAG00000052665	4.01	9.7 × 10^−14^	cytochrome P450, subfamily IIIA, polypeptide 4	CYP3A4 CYP3A28	**↑**
ENSBTAG00000016906	5.43	4.3 × 10^−13^	cytochrome P450, family 27, subfamily B, polypeptide 1	CYP27B1	**↑**
ENSBTAG00000010531	−1.87	4.4 × 10^−8^	cytochrome P450, family 1, subfamily B, polypeptide 1	CYP1B1	**↓**
ENSBTAG00000012972	−1.39	1.3 × 10^−6^	cytochrome P450, family 2, subfamily U, polypeptide 1	CYP2U1	**↓**
ENSBTAG00000014890	−5.61	1.8 × 10^−6^	cytochrome P450, family 19, subfamily A, polypeptide 1	CYP19A1	**↓**
ENSBTAG00000003632	−1.91	3.0 × 10^−6^	cytochrome P450, family 39, subfamily A, polypeptide 1	CYP39A1	**↓**
ENSBTAG00000039319	−2.09	7.3 × 10^−6^	cytochrome P450, family 4, subfamily F, polypeptide 2	CYP4F2	**↓**
ENSBTAG00000011976	−5.65	1.0 × 10^−5^	cytochrome P450, family 4, subfamily B, polypeptide 1	CYP4B1	**↓**
ENSBTAG00000053766	−3.24	7.7 × 10^−5^	cytochrome P450 2J2-like	CYP2J2L	**↓**
ENSBTAG00000003871	−3.31	0.00031	cytochrome P450 subfamily 2B	CYP2B6	**↓**
ENSBTAG00000001021	−1.28	0.00064	cytochrome P450 family 1 subfamily A member 1	CYP1A1	**↓**

**Table 5:** Gene expression (↑, upregulation; ↓, downregulation) of GST isoenzymes after AFB1 treatment. For each DEG (AFB1 vs PCB126), Ensembl gene ID, log_2_ fold change (logFC), and False Discovery Rate (FDR) are provided as reported in edgeR output. The Ensembl gene description and acronym are also provided.

Ensembl Gene ID	logFC	FDR	Gene Description	Gene Acronym	Pattern of Expression
ENSBTAG00000008541	−3.49	8.4 × 10^−15^	microsomal glutathione S-transferase 1	MGST1	**↓**
ENSBTAG00000000170	−2.67	4.9 × 10^−11^	glutathione S-transferase, theta 4	GSTT4	**↓**
ENSBTAG00000017765	−1.76	1.1 × 10^−10^	Bos taurus glutathione S-transferase M1	GSTM1	**↓**
ENSBTAG00000006546	−2.85	6.4 × 10^−8^	glutathione S-transferase alpha 2	GSTA2	**↓**
ENSBTAG00000002706	−1.33	2.3 × 10^−6^	glutathione S-transferase zeta 1	GSTZ1	**↓**
ENSBTAG00000040298	−1.11	1.0 × 10^−5^	glutathione S-transferase theta 1	GSTT1	**↓**
ENSBTAG00000003989	1.07	4.6 × 10^−5^	glutathione S-transferase omega 1	GSTO1	**↑**
ENSBTAG00000010265	−0.83	0.00255	microsomal glutathione S-transferase 3	MGST3	**↓**
ENSBTAG00000038540	−1.54	0.00308	glutathione S-transferase omega-1	GSTO1	**↓**
ENSBTAG00000051210	0.72	0.00626	glutathione S-transferase pi 1	GSTP1	**↑**
ENSBTAG00000003548	1.13	0.00796	glutathione S-transferase pi 1	GSTP1	**↑**
ENSBTAG00000037673	−0.99	0.00799	glutathione S-transferase mu 1	GSTM1	**↓**
ENSBTAG00000021779	−0.77	0.01075	microsomal glutathione S-transferase 2	MGST2	**↓**

**Table 6:** Enriched hallmark Gene Sets (GSs) obtained from Gene Set Enrichment Analysis (GSEA) of transcripts up- (top) and downregulated (bottom) by AFB1 in BFH12 cells. ES: enrichment score; NES: normalized enrichment score; NOM *p*-val: nominal *p*-value; FDR: False Discovery Rate.

**Hallmark GS Upregulated by AFB1**	**GS Size**	**ES**	**NES**	**NOM *p*-val**	**FDR *q*-val**
MYC_targets_v1	196	0.39	6.20	0.000	0.000
TNFα_signaling_via_NF-kB	174	0.34	5.23	0.000	0.000
Oxidative_phosphorylation	194	0.32	5.18	0.000	0.000
MYC_targets_v2	55	0.56	4.83	0.000	0.000
E2F_targets	195	0.23	3.79	0.000	0.000
DNA_repair	143	0.25	3.58	0.000	0.000
G2M_checkpoint	192	0.22	3.53	0.000	0.000
Inflammatory_response	121	0.24	3.04	0.000	0.000
UV_response_up	133	0.19	2.58	0.000	0.000
KRAS_signaling_up	141	0.18	2.50	0.000	0.000
Unfolded_protein_response	108	0.19	2.27	0.000	0.003
Allograft_rejection	102	0.17	1.97	0.010	0.013
MTORC1_signaling	192	0.12	1.96	0.002	0.013
P53_pathway	184	0.12	1.84	0.015	0.022
Interferon gamma_response	139	0.13	1.73	0.024	0.038
IL2_STAT5_signaling	158	0.11	1.61	0.030	0.063
PI3K_AKT_mTOR_signaling	92	0.14	1.60	0.035	0.063
Apoptosis	135	0.12	1.59	0.047	0.062
Hypoxia	176	0.10	1.58	0.047	0.064
**Hallmark Gene Sets Downregulated by AFB1**	**Size**	**ES**	**NES**	**NOM *p*-val**	**FDR *q*-val**
Epithelial_mesenchymal_transition	168	−0.25	−3.74	0.000	0.000
UV_response_dn	128	−0.23	−3.06	0.000	0.000
Protein_secretion	91	−0.25	−2.77	0.000	0.001
Apical_junction	156	−0.17	−2.59	0.000	0.001
Myogenesis	141	−0.18	−2.59	0.000	0.001
KRAS_signaling_dn	93	−0.20	−2.31	0.000	0.003
Coagulation	90	−0.19	−2.16	0.002	0.007
Apical_surface	31	−0.29	−1.95	0.010	0.020
Bile_acid_metabolism	76	−0.19	−1.92	0.004	0.022
Estrogen_response_late	145	−0.13	−1.82	0.022	0.035
Estrogen_response_early	154	−0.12	−1.81	0.014	0.034
Angiogenesis	26	−0.28	−1.70	0.032	0.053
Heme_metabolism	154	−0.11	−1.60	0.042	0.083

## Supplementary Materials

The following are available online at https://www.mdpi.com/2072-6651/12/7/429/s1, Figure S1: Real-time PCR transcriptional changes induced by PCB126, Figure S2: Modulation of AFB1 cytotoxicity in the presence of the AHR agonist PCB126, Figure S3: MDS plot, Figure S4: Real-time PCR transcriptional changes induced by AFB1, Table S1: Sequencing and mapping results, Table S2: Differential expression analysis, Table S3: Functional enrichment, Table S4: Real-time PCR assays, File S1. EdgeR analysis.
